# Negative Phonotaxis Behavior of Juvenile Grass Carp (*Ctenopharyngodon idella*) to Different Acoustic Stimuli in Natural Aquatic Environments

**DOI:** 10.3390/ani16091401

**Published:** 2026-05-03

**Authors:** Jiaxin Li, Shenwei Zhang, Xuan Wang, Ji Yang, Guoyong Liu, Lixiong Yu

**Affiliations:** 1 Hubei International Science and Technology Cooperation Base of Fish Passage, China Three Gorges University, Yichang 443002, China; li18271896573@163.com (J.L.); 13515722852@163.com (S.Z.);; 2 Yangtze River Fisheries Research Institute, Chinese Academy of Fishery Science, National Agricultural Science Observing and Experimental Station of Chongqing, Wuhan 430233, China; 3 College of Hydraulic and Environment Engineering, China Three Gorges University, Yichang 443002, China; 4 China Gezhouba Group No. 3 Engineering Co., Ltd., Xi’an 710077, China; yangji0718@163.com

**Keywords:** acoustic barriers, impulsive sound, negative phonotaxis behavior, *Alligator sinensis* hissing, pile-driving noise, outboard motor sound, *Ctenopharyngodon idella*

## Abstract

Hydraulic engineering structures can threaten freshwater fish by entraining them into hazardous areas. Acoustic barriers have been proposed as a non-physical method to guide fish away from these zones. In this study, we investigated the behavioral responses of juvenile grass carp to different acoustic stimuli under semi-natural conditions using outdoor net cages. Four sound types were tested: a 1000 Hz pure tone and three broadband sounds, including *Alligator sinensis* hissing, pile-driving noise, and outboard motor noise. Behavioral responses were quantified using response frequency, total midline crossings, first-response time, maximum swimming speed, and average swimming speed. The results showed that *Alligator sinensis* hissing elicited the highest number of midline crossings, representing the strongest behavioral response among all tested sounds. In addition, both *Alligator sinensis* hissing and outboard motor noise induced significantly stronger avoidance responses than the pure tone or pile-driving noise, as indicated by higher response frequency and faster swimming speeds. Furthermore, manipulation of pulse repetition intervals in the most effective deterrent sounds generated a novel broadband sound, which altered fish distribution patterns and elicited avoidance behavior. These findings indicate that both sound type and temporal structure influence negative phonotaxis behavior in grass carp and provide experimental evidence for the optimization of acoustic barriers in fish management.

## Simple Summary

Hydraulic engineering structures can unintentionally draw freshwater fish into hazardous areas, posing risks to their safety. Acoustic barriers provide a non-physical method to guide fish away from such zones. However, previous experiments were primarily conducted in enclosed tanks or flumes, where sound propagation characteristics differ substantially from those in natural waters (e.g., particle motion of sound and frequency distortion caused by ambient noise), particularly due to the absence of tank wall reflections, standing wave formation, and the simplified acoustic field associated with limited boundaries and reduced multipath propagation. As a result, the effectiveness of these acoustic stimuli under field conditions remains largely unverified. In this study, we investigated the behavioral responses of grass carp (*Ctenopharyngodon idella*) to different sounds in natural outdoor net cages. The tested sounds included a 1000 Hz pure tone and three broadband sounds: *Alligator sinensis* hissing, pile-driving noise, and outboard motor noise. Fish behavior was quantified in terms of response frequency and swimming speed. The results showed that *Alligator sinensis* hissing and outboard motor noise elicited the strongest avoidance behavior, and different pulse repetition intervals of the most effective deterrent sounds also influenced fish distribution within the experimental area. These findings indicate that both sound type and temporal structure affect fish behavior and provide valuable information for designing effective acoustic deterrent systems.

## 1. Introduction

Hydraulic engineering projects, such as hydropower stations and agricultural irrigation systems, can generate substantial socioeconomic benefits by regulating water resources for electricity production and crop irrigation. However, these projects may also exert significant negative impacts on aquatic ecosystems [1,2] and freshwater fish populations [3]. In particular, dam construction can obstruct the upstream migration of migratory fish, while water diversion structures and pumping operations may result in injury or mortality of both juvenile and adult fish [4]. To mitigate this issue, various mitigation measures have been implemented, including the deployment of physical barriers to prevent fish from entering hazardous areas. Although physical barriers can reduce fish entrainment to some extent [5], they are often associated with high construction costs [6] and practical challenges in installation and maintenance [7]. Consequently, acoustic deterrent systems have been increasingly adopted as non-physical barriers that can deter fish without causing physical harm, and have been widely applied at ship locks, dam facilities, water intake channels, and other strategic locations [8]. For instance, Riesgraf et al. (2022) [9] deployed an acoustic deterrent system at Lock and Dam 8 (LD8) on the Mississippi River to prevent common carp from entering the lock. In addition, these systems are utilized to prevent migrating fish from entering power plants [10]. These systems emit specific acoustic signals that interfere with fish behavior, thereby creating an acoustic barrier that prevents fish from entering restricted areas [11]. Previous studies have tested various sound types, such as outboard motor noise and predator sounds (e.g., *Alligator sinensis* hissing), to evaluate their effects on fish behavior under indoor laboratory conditions [12,13]. However, these experiments were primarily conducted in enclosed tanks or flumes, where sound propagation characteristics differ substantially from those in natural waters [14]. As a result, the effectiveness of these acoustic stimuli under field conditions remains largely unverified. Therefore, it is necessary to screen and evaluate acoustic stimuli for target species and further validate their behavioral effects in more natural environments [15].

Sliver carp (*Hypophthalmichthys molitrix*) and big head carp (*Aristichthys nobilis*) exhibit negative phonotaxis behavior to outboard motor noise within the frequency range of 0–10 kHz [12,16]. Similarly flower fish and tu-fish (*Schizopygopsis younghusbandi*) exhibit avoidance of sound sources when exposed to *Alligator sinensis* hissing within the frequency range of 0.05–5 kHz [13,17]. These findings suggest that broadband sounds can induce avoidance behaviors in fishes [12]. Conversely, at a water temperature of 18 ± 2 °C, common carp (*Cyprinus carpio*) exhibited no avoidance of sound sources when exposed to outboard motor noise within the frequency range of 60–10 kHz [18]. In addition, at water temperatures of 20–25 °C, grass carp exhibited a certain level of deterrence to a 100 Hz pure tone [19]. However, these studies have often screened sounds to which fish are sensitive in indoor enclosed tanks, which has hindered the development of acoustic deterrent systems and raised doubts about their effectiveness. In indoor tank experiments, sound propagation is affected by reflections from boundaries (e.g., glass walls or the water surface), which can generate standing waves [14]. The presence of standing waves may lead to localized resonance and uneven sound pressure level distributions within the sound field [14]. Consequently, few studies have examined the effects of acoustic stimuli on fish behavior under natural or semi-natural aquatic conditions. In addition to sound pressure, fish are capable of detecting the particle motion component of acoustic signals, which plays a particularly important role in auditory perception. Particle velocity is especially relevant in shallow-water and near-field environments, where it may dominate over sound pressure as the primary cue for sound detection [20,21]. Many fish species, including cyprinids such as grass carp, possess a Weberian apparatus that enhances their sensitivity to acoustic stimuli by improving the detection of pressure fluctuations [22,23]. For instance, *H. molitrix* and *A. nobilis* are capable of detecting sounds within the frequency range of 100–3000 Hz [12,16]. However, their behavioral responses are still strongly influenced by particle motion, particularly at low frequencies, where auditory thresholds are generally lower [24]. For instance, low-frequency sounds at 70 Hz or 90 Hz were effective in inhibiting the migration of sea lampreys (*Petromyzon marinus*) [25].

It is well established that assessing the impacts of underwater noise is complex, in part because anthropogenic noise exhibits considerable variability in amplitude, frequency spectrum, and temporal patterns [26,27]. Among these characteristics, the temporal structure of sound has received relatively little attention, despite its potential importance in eliciting behavioral responses in fish [28,29]. For instance, Neo et al. (2014) [27] reported that European seabass (*Dicentrarchus labrax*) exhibited consistent initial behavioral responses to noise exposure, but recovered more slowly from impulsive sounds than from continuous sounds. Sabet et al. (2015) [30] reported that exposure of zebrafish to four different temporal patterns of sound—including continuous, fast- and slow-regular intermittent, and irregular intermittent—significantly increased startle responses and swimming speed. Given that impulsive sounds vary in multiple temporal characteristics, there is a need for systematic investigations of additional temporal parameters, including pulse repetition interval, pulse repetition regularity, pulse duration, and pulse shape (e.g., rise time) [31]. However, it remains unclear whether pulse repetition interval (PRI) also affects the avoidance responses of fish to impulsive sound exposures.

The present study conducted sound playback experiments in natural water environments to investigate the behavioral responses of juvenile grass carp to three types of broadband sounds and a 1000 Hz pure tone. The aim was to compare behavioral responses in natural open-water conditions with those observed in previous laboratory experiments, thereby assessing potential differences in avoidance behavior. In addition, the distribution rate and the selection coefficient of juveniles were quantified to evaluate their avoidance responses to different PRI sounds. The results of this study provide empirical evidence to support the further development of acoustic deterrent systems.

## 2. Materials and Methods

### 2.1. The Experimental Fish

A total of 200 juvenile grass carp with a body length of 13.56 ± 1.23 cm (mean ± SD) and body mass of 38.45 ± 2.89 g (mean ± SD) were obtained from the national original breeding farm of four major Chinese carps (FMCCs) in Jingzhou City, Hubei Province, China. The experimental fish were transported from the breeding base to the aquaculture facility at Qixin Reservoir (30°22′ N, 114°18′ E) using fish transport trucks equipped with an oxygenation system, and were temporarily held in three recirculating tanks (1800 L). Aquaculture tanks employed recirculating water sourced from the Qixin reservoir in Wuhan. A thermostatic system maintained water temperature at 25.0 ± 0.5 °C, and dissolved oxygen was kept above 7.0 mg/L. Juvenile grass carp were fed phytoplankton-based feed once daily in the evening. After 1 h of feeding, uneaten feed and feces were removed using a siphon.

### 2.2. Experimental Arena

**Figure 1:**
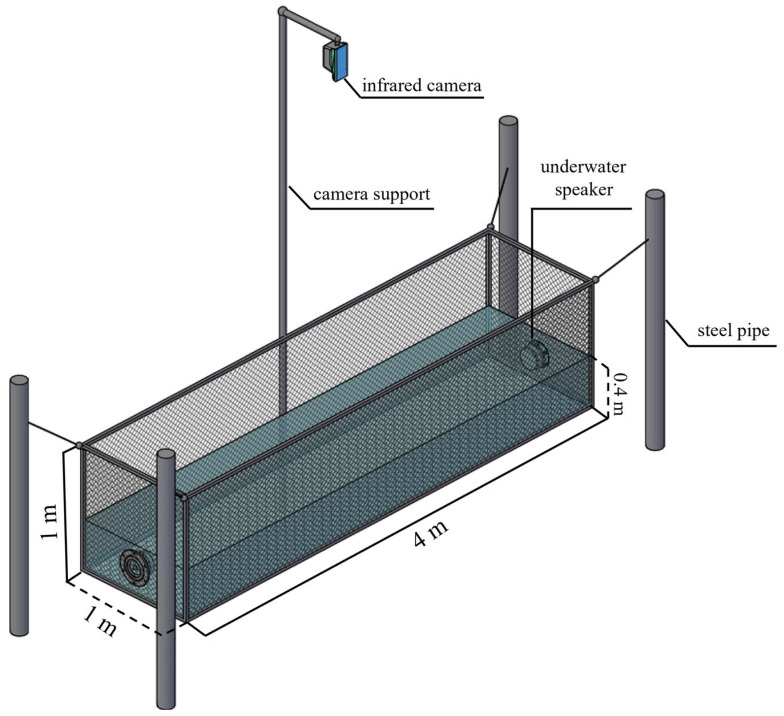
A view of experimental cage setup.

Behavioral experiments were conducted in an outdoor net cage (4 × 1 × 1 m; length × width × depth) with a mesh size of 0.7 cm (Figure 1), which prevented the experimental fish from escaping and did not interfere with sound transmission. A pulley system was used to lower the net cage into the reservoir at a distance of 1.0 m from the shore, and the cage was secured in place using four steel pipes, each 3.0 m in length. The water depth within the cage was maintained at 0.4 m. All experiments were conducted at night to maintain a relatively constant water temperature and to eliminate potential adverse effects of sunlight on fish behavior. To facilitate observation of negative phonotaxis behavior in juvenile grass carp, an infrared camera (DS-3WF01C-2NE, Hikvision, Hangzhou, China) was positioned 4.0 m directly above the net cage to record the experiments.

### 2.3. Sound Stimuli

#### 2.3.1. Pure Tone and Broadband Sound

1. 1000 Hz pure tone

Generated by Adobe Audition 2020 (Adobe Inc., San Jose, CA, USA). All selected 1000 Hz pure tones had a duration of 4 min.

2. *Alligator sinensis* hissing

*Alligator sinensis* hissing was recorded at the Anhui Research Center for Chinese Alligator Reproduction (ARCCAR) using a digital recorder (Nagra SD; 48 kHz sampling rate, 16-bit resolution; Nagra, Wettingen, Switzerland), following the methods described by Liu et al. (2018) [16]. Briefly, a calibrated hydrophone (Reson TC 4032; Slangerup, Denmark; sensitivity −170 dB re 1 V/μPa; frequency range 5 Hz–120 kHz) was positioned underwater at a depth of 0.1 m, mounted at one end of a 2.0 m steel pole, and connected to a battery-powered digital audio tape recorder. All vocalizations were recorded at an approximate distance of 1.5 m from the vocalizing individuals. Signals with high signal-to-noise ratios and representative hissing characteristics were selected as playback stimuli. All selected hissing signals had a duration of 4.8 s.

3. Pile-driving noise

Pile-driving noise was recorded at a construction site along the riverbank in Xiling District, Yichang City. The recordings were made using a digital recorder. Briefly, a calibrated hydrophone was positioned underwater at a depth of 0.1 m, mounted at one end of a 3.0 m bamboo pole, and connected to a battery-powered digital audio tape recorder. Signals with high signal-to-noise ratios were selected as playback stimuli. All selected pile-driving signals had a duration of 8 s.

4. Outboard motor noise

Outboard motor noise was recorded while the Yangtze River cruise was traveling at a speed of 25 km/h. Briefly, a hydrophone was positioned 3.5 m away from the cruise and suspended 0.3 m below the water surface. Signals with high signal-to-noise ratios were selected for subsequent playback experiments. All selected outboard motor signals had a duration of 9 s.

All recordings were converted to the WAV format and stored on a computer using Adobe Audition (v2020, Adobe Inc., San Jose, CA, USA) software. Playback experiments were conducted according to Liu et al. (2018) [13], Two Pairs of underwater speakers (UWS-045, Khz Electronic Technology Co., Ltd., Zhongshan, China; effective frequency range 80–18,000 Hz) were positioned at each end of the cage, with one pair of speakers at each end, and alternately delivered the experimental sound stimuli, which included a 1000 Hz pure tone and broadband sounds. To minimize vibration, the speakers were placed 0.5 m from the bottom and 0.5 m from the sidewalls of the cage; each pair of speakers was hung from a string 0.2 m above the cage and attached to two iron poles located on the left and right sides of the cage.

#### 2.3.2. Impulsive Sounds

1. Impulsive hissing sounds of *Alligator sinensis*

Audio processing of *Alligator sinensis* hissing was conducted using Adobe Audition 2020. Five playback conditions were generated as follows:

(a) Condition AH consisted of continuous *Alligator sinensis* hissing.
(b) Condition BH consisted of *Alligator sinensis* hissing with a PRI of 1.5 s.
(c) Condition CH consisted of *Alligator sinensis* hissing with a PRI of 2 s.
(d) Condition DH consisted of *Alligator sinensis* hissing with a PRI of 3 s.
(e) Condition EH consisted of *Alligator sinensis* hissing with a PRI of 5 s.
(f) Condition FH was a random intermittent noise, composed of 1 s *Alligator sinensis* hissing alternating with silent intervals of random duration ranging from 1 to 7 s, with a mean silent interval of approximately 4 s. Due to the random variation in silent interval duration, this condition did not have a fixed PRI.

2. Impulsive sounds of outboard motor noise

Audio processing of outboard motor noise was conducted using Adobe Audition 2020. Five playback conditions were generated as follows:

(a) Condition AS consisted of continuous outboard motor noise.
(b) Condition BS consisted of outboard motor noise with a PRI of 5 s.
(c) Condition CS consisted of outboard motor noise with a PRI of 2 s.
(d) Condition DS consisted of outboard motor noise with a PRI of 1.5 s.
(e) Condition ES was a random intermittent noise, composed of 1 s of outboard motor noise alternating with silent intervals of random duration ranging from 1 to 7 s, with a mean silent interval of approximately 4 s. This condition did not have a fixed PRI due to random variation in silent interval duration.

Playback experiments were conducted according to Neo et al. (2015) [31], with some modifications. A single underwater speaker was placed at one end of the net cage and used to play the experimental sound stimuli. The speaker was positioned 0.5 m above the cage bottom and 0.5 m from the sidewall of the cage. To minimize vibration, the speaker was suspended from a string 0.2 m above the cage. It was attached to two iron poles located on the left and right sides of the cage.

All tested sounds were amplified with a power amplifier (XLS-1000, Crown Audio, Inc., Tokyo, Japan); the sound intensities were manually adjusted using the switch on the amplifier. The sounds were broadcast from a laptop (15-cx0059TX, Hewlett-Packard, Palo Alto, CA, USA) via Adobe Audition 2020. The acoustic characteristics of the speakers within the cage were measured using a Reson TC 4032 hydrophone connected to a sound level meter (AWA6291, Hangzhou Aihua Instruments Co., Ltd., Hangzhou, China), based on the equivalent sound level measured over 15 s. To map the acoustic field, the cage was divided into a 0.1 × 0.1 m grid. In addition, the acoustic recordings were manually logged and plotted using Origin 2024 (64-bit, OriginLab Corporation, Northampton, MA, USA) software.

Particle motion may be dominant in fish hearing [32], but it could not be measured in this study. However, the absence of such information does not affect our objectives, as our goal is not to determine absolute threshold levels for extrapolation to natural conditions. Rather, we aim to compare whether fish selectively respond to sounds under natural versus laboratory conditions while controlling for other acoustic parameters, and to assess the effects of PRI on behavioral responses.

### 2.4. Experimental Set-Up

We tested one hundred and seventy fish: sixty in the experiments of pure tone and broadband sounds, and one hundred and ten in the experiment of impulsive sounds. The experiments were carried out from 1 June to 20 July 2024.

#### 2.4.1. Pure Tone and Broadband Sounds Experimental Methods

The experiments were conducted according to Liu et al. (2018) [13], with minor modifications. The experiment consisted of a control group and an experimental group. In the control group, no sound was played, whereas sound stimuli were presented in the experimental group. Based on previous studies, the duration of both the control and experimental trials was set to 5 min. At the beginning of each trial, a juvenile grass carp was randomly selected from the holding tank and placed into the experimental cage, where it was allowed to acclimate for 1 h. After acclimation, the control group trial was initiated, during which the behavior of the fish was recorded using an infrared camera with no sound playback. For the experimental group, the position of the test fish was first observed. When the fish approached one side of the cage near an underwater speaker, the power amplifier was manually switched on to initiate sound playback. If the fish moved away from the sound source within 15 s and crossed the centerline of the cage (2 m) within 30 s, sound playback on that side was immediately stopped and the underwater speaker on the opposite side was activated. If this behavior did not occur, sound playback was maintained on the same side until the end of the trial. During sound trials, the order of sound exposure was randomized to minimize potential “carry-over” effects due to sequential exposures. An observer operated the sound unit from a tent located approximately 3 m from the cage, while an infrared camera was set up to video record the observer’s operation. During the experiment, the observer remained silent to prevent interference. In addition, a hydrophone was used to record and examine sound artifacts when the underwater speakers were turned on and off, ensuring that only the experimental acoustic stimuli were recorded. Trials were conducted between 24:00 and 04:30, and each experimental condition was repeated 10 times. After each trial, an untested, healthy, and active fish was selected from the holding tank and introduced into the cage to begin the next trial.

#### 2.4.2. Impulsive Sounds Experimental Methods

**Figure 2:**
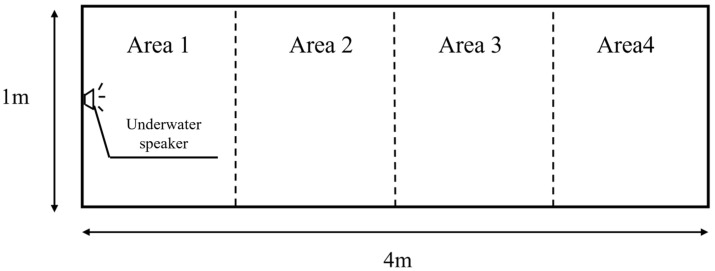
Schematic diagram of the impulsive sound experiment.

The experiments were conducted according to Neo et al. (2015) [31], with minor modifications. The experiment consisted of a control group and an experimental group. In the control group, continuous *Alligator sinensis* hissing and an outboard motor were used, whereas the experimental group was exposed to impulsive sounds with different PRIs. The duration of both the control and experimental trials was set to 5 min. At the beginning of each trial, a juvenile grass carp was randomly selected from the holding tank and placed into Area 1 of the cage, where it was allowed a 1 h acclimation period. After the acclimation period, the nylon net separating Area 1 and Area 2 was slowly lifted, after which impulsive sounds were played using the underwater speaker (Figure 2). During the trials, the order of sound exposure was randomized to minimize potential “carry-over” effects resulting from sequential exposures. There was no external anthropogenic noise or disturbance in the vicinity of the study area during the trials. Trials were conducted between 23:00 and 05:00, and each experimental condition was repeated 10 times. After each trial, the tested fish was replaced with a new healthy, untested individual.

#### 2.4.3. Intensity of Tested Sound

The intensity of all tested sounds was increased to 115 dB re 1 μPa by adjusting the switch on the power amplifier. These measurements were made with a hydrophone (Reson TC 4032; Slangerup, Denmark; sensitivity −170 dB re 1 V/μPa; frequency range 5 Hz–120 kHz) connected to a sound level meter (AWA6291, Hangzhou Aihua Instruments Co., Ltd., Hangzhou, China).

### 2.5. Data Analysis

#### 2.5.1. Pure Tone and Broadband Sounds Trial

**Figure 3:**
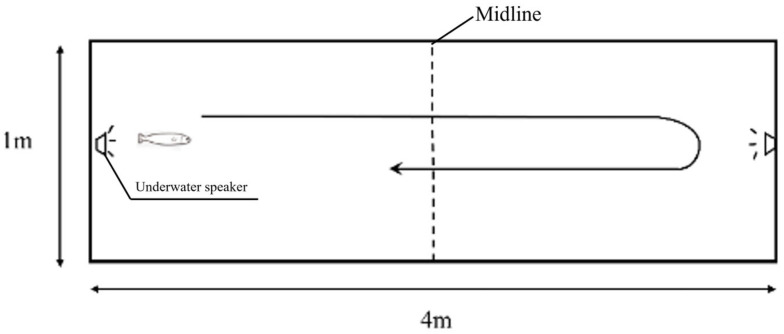
Diagram Illustrating Negative Phonotaxis.

1. Negative Phonotaxis Behavioral: A negative phonotactic response was defined as juvenile grass carp swimming away from the end area closest to the active speaker pair within 15 s and crossing the centerline (2 m) of the net cage within 30 s after unilateral sound playback was initiated (Figure 3). No response and consecutive responses were defined following Vetter et al. (2015) [12]. Specifically, all behaviors not meeting the predefined criteria of a negative phonotactic response—such as swimming toward the active speaker pair, swimming as observed in control fish, or crossing the midline after more than 30 s were classified as no response. Consecutive responses were defined as successive reactions, each complying with the predefined criteria of a negative phonotactic response, occurring after two or more successive sound exposures.
2. Response frequency: the total number of negative phonotactic behaviour exhibited.
3. Total midline number of crossings: the total number of back-and-forth crossings of the cage centerline by juvenile grass carp during the 5 min experiment.
4. First response time: the time from the onset of sound playback to the first crossing of the midline by juvenile grass carp.
5. Maximum swimming speed: the peak swimming speed exhibited by the experimental fish during the 5 min experiment after sound playback.
6. Average response speed: the swimming speed of juvenile grass carp during each back-and-forth crossing of the cage centerline was recorded as the response speed, and the mean of all such speeds was defined as the average response speed.

#### 2.5.2. Impulsive Sounds Trial

1. 1. Distribution rate (*F*): the proportion of total time that juvenile grass carp spent in each experimental area.

(1) $$F=\frac{t}{T}\times 100\%$$

In the equation, *t* denotes the time juvenile grass carp spent in a given area of the cage, and *T* denotes the total duration of the experiment.

2. The selection coefficient (*E*) represents the selectivity of juvenile grass carp for different impulsive sound stimuli.

(2) $$E=\frac{f-P}{P}$$

In the equation, *E* is the selection index, where *E* = 0 indicates no selection, *E <* 0 indicates avoidance, and *E* > 0 indicates preference. A larger absolute value of *E* reflects a stronger phonotactic tendency toward a given sound. *f* denotes the proportion of experimental fish distributed in a given area under a specific sound, and *P* represents the proportion of all experimental fish occurring in that area, calculated as the time spent in the area divided by the total experimental duration.

#### 2.5.3. Statistical Analysis

Tracker (version 5.0.5, Open Source Physics, Geneva, Switzerland) was used to determine the location of grass carp in two dimensions while they moved. The consecutive frames (25 fps/s) of the recorded video were analyzed, and the swimming speed of an individual fish was extracted by connecting its positional coordinates in chronological order. The experimental data were preprocessed using Microsoft Excel 2018. All tested sounds were analyzed using fast Fourier transform (FFT) in Adobe Audition 2020. Impulsive sounds were transformed with an FFT size of 4096, whereas other tested sounds were analyzed with an FFT size of 2048 (sampling rate: 44.1 kHz). The resulting frequency spectra and sound field maps were generated using Origin 2024.

All statistical analyses were performed in R 4.4.3. The *Shapiro–Wilk* test was used to assess normality and homogeneity of variance. Response frequency, total midline number of crossings, and average response speed did not meet the assumptions of normality; therefore, the *Kruskal–Wallis* test was applied, followed by pairwise comparisons with *Bonferroni* correction to determine significant differences among groups. In contrast, first response time, maximum swimming speed, and distribution rate (*F*) satisfied the assumptions of normality and homogeneity of variance, and a one-way ANOVA was used to test for significant differences. Regression analyses were conducted to fit polynomial models and evaluate the selectivity of juvenile grass carp to different impulsive sounds. Differences were considered statistically significant at *p* < 0.05.

## 3. Results

### 3.1. Characteristics of Sound

**Figure 4:**
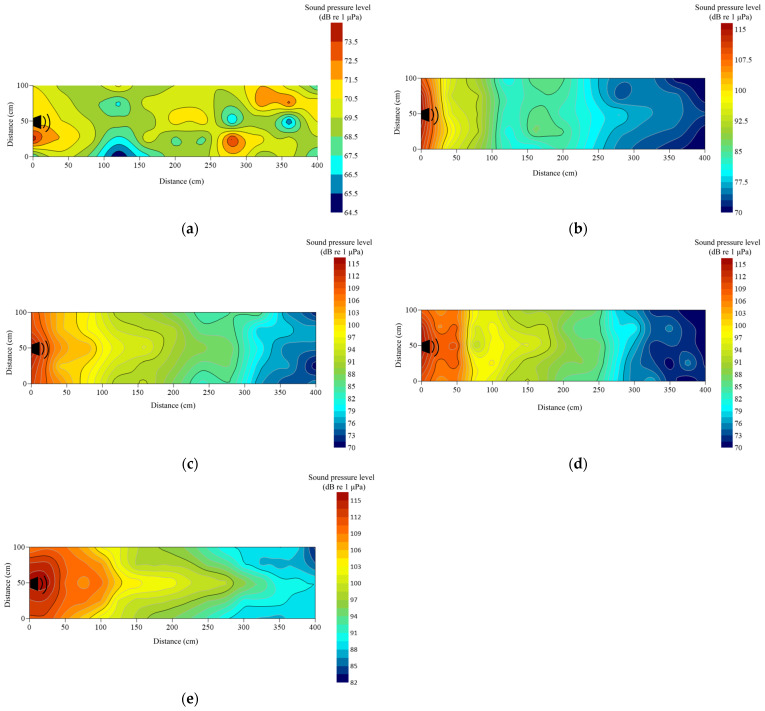
Contour maps of sound pressure level (dB re 1 μPa) in the experimental cage. The measurement of sound intensity was made at a depth of 0.2 m at 400 positions in the cage during the playback of the sound stimulus. The intensity level was represented by different colors, marked on the scale on the right: (**a**) ambient; (**b**) 1000 Hz pure tone; (**c**) pile-driving noise; (**d**) outboard motor noise; (**e**) *Alligator sinensis* hissing.

Under no-playback conditions, underwater sound pressure levels (SPL) ranged from 65 to 75 dB re 1 μPa and were spatially heterogeneous (Figure 4a). Activation of the underwater loudspeaker produced a maximum SPL of approximately 115 dB re 1 μPa near the source. For the 1000 Hz pure tone, SPL decreased to 70 dB at the end of the cage, yielding a total attenuation of 45 dB re 1 μPa (Figure 4b). Pile-driving noise exhibited weak longitudinal attenuation but rapid lateral decay within 1 m of the loudspeaker; terminal SPL was 75 dB re 1 μPa, corresponding to 40 dB re 1 μPa attenuation (Figure 4c). The outboard motor showed a comparable lateral decay pattern, with pronounced attenuation at 1.2 m and an overall reduction of 38.48 dB re 1 μPa (Figure 4d). In contrast, *Alligator sinensis* hissing attenuated more gradually, with lateral reduction becoming evident at 2.0 m; terminal SPL remained relatively high (~88 dB re 1 μPa), and total attenuation was 27 dB re 1 μPa (Figure 4e). Overall, background noise and the 1000 Hz tone maintained lower SPLs. Pile-driving and outboard motor noise demonstrated similar lateral attenuation profiles characterized by rapid near-source decay, whereas the alligator roar exhibited slower lateral attenuation. Longitudinal attenuation was generally limited across treatments.

**Figure 5:**
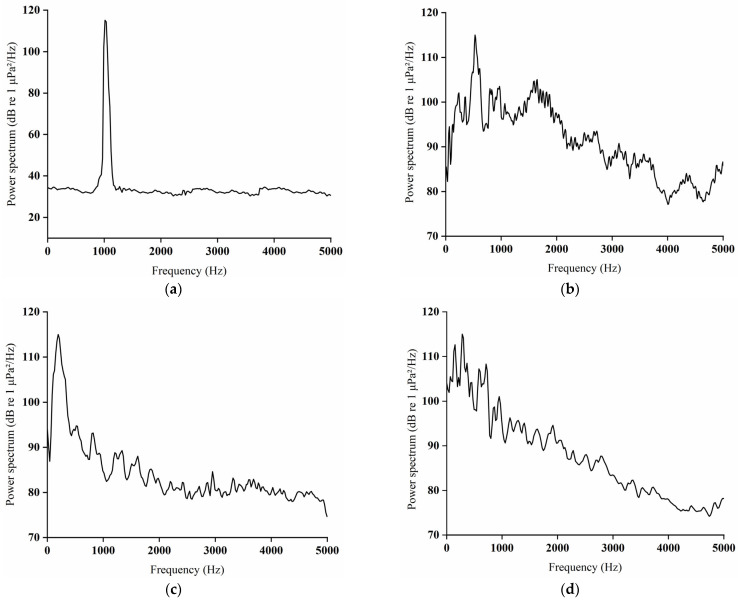
The power spectrum: (**a**) 1000 Hz pure tone; (**b**) pile-driving noise; (**c**) *Alligator sinensis* hissing; (**d**) outboard motor noise.

The 1000 Hz pure tone exhibited an overall frequency distribution spanning 0–5000 Hz, with a dominant spectral peak at 1000 Hz (Figure 5a). Pile-driving noise also covered a frequency range of 0–5000 Hz, showing a pronounced spectral peak between 400 and 600 Hz (Figure 5b). *Alligator sinensis* hissing was distributed across 0–5000 Hz, with major spectral energy concentrated between 100 and 400 Hz; beyond 3500 Hz, the spectrum remained relatively stable with no additional prominent peaks (Figure 5c). In contrast, outboard motor noise displayed peak spectral energy within the low-frequency band of 50–500 Hz (Figure 5d).

**Figure 6:**
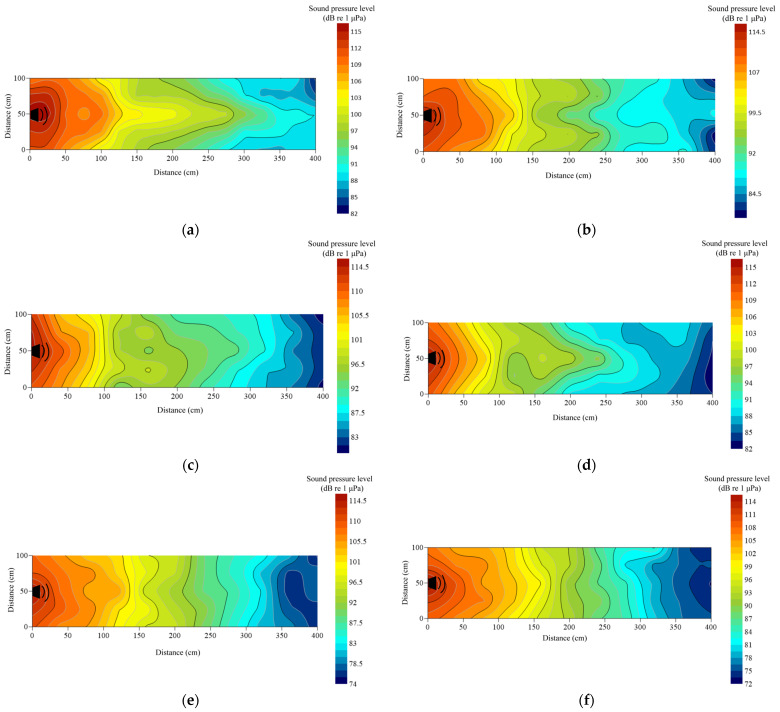
Contour maps of sound pressure level (dB re 1 μPa) of *Alligator sinensis* hissing with different PRIs. The measurement of sound intensity was made at a depth of 0.2 m at 400 positions in the cage during the playback of the sound stimulus. The intensity level was represented by different colors, marked on the scale on the right: (**a**) AH; (**b**) BH; (**c**) CH; (**d**) DH; (**e**) EH; (**f**) FH.

*Alligator sinensis* hissing signals with different PRIs exhibited some differences in the spatial distribution of sound pressure levels. During playback of AH, the overall attenuation was approximately 27 dB re 1 μPa, indicating a gradual decrease in sound level during propagation (Figure 6a). For BH, CH, and DH, the minimum SPL measured at the end of the experimental net cage ranged from 80 to 86 dB, with total attenuation between 29 and 35 dB re 1 μPa (Figure 6b–d). Although the overall attenuation magnitudes were similar, the spatial patterns of sound pressure distribution varied across positions. During playback of EH, the maximum SPL near the loudspeaker reached 115 dB, while the minimum SPL at the terminal end was 75.21 dB re 1 μPa, yielding the greatest attenuation (~40 dB re 1 μPa) (Figure 6e). During playback of FH, the minimum SPL at the end of the cage was 73.27 dB re 1 μPa (Figure 6f).

The observed differences should not be attributed to PRI itself, as sound attenuation in water is primarily governed by geometric spreading, medium absorption, and boundary conditions, rather than the pulse repetition interval. Variations in SPL distribution are more likely associated with differences in the temporal structure and spectral characteristics of the signals, as well as their interactions with the experimental environment. In addition, boundary reflections, scattering from cage structures, and environmental heterogeneity under open-water conditions may further contribute to spatial variability in the sound field. Overall, all *Alligator sinensis* hissing signals exhibited relatively gradual attenuation along the longitudinal direction, whereas attenuation in the lateral direction was more rapid, reflecting the combined influence of acoustic propagation conditions and signal properties.

**Figure 7:**
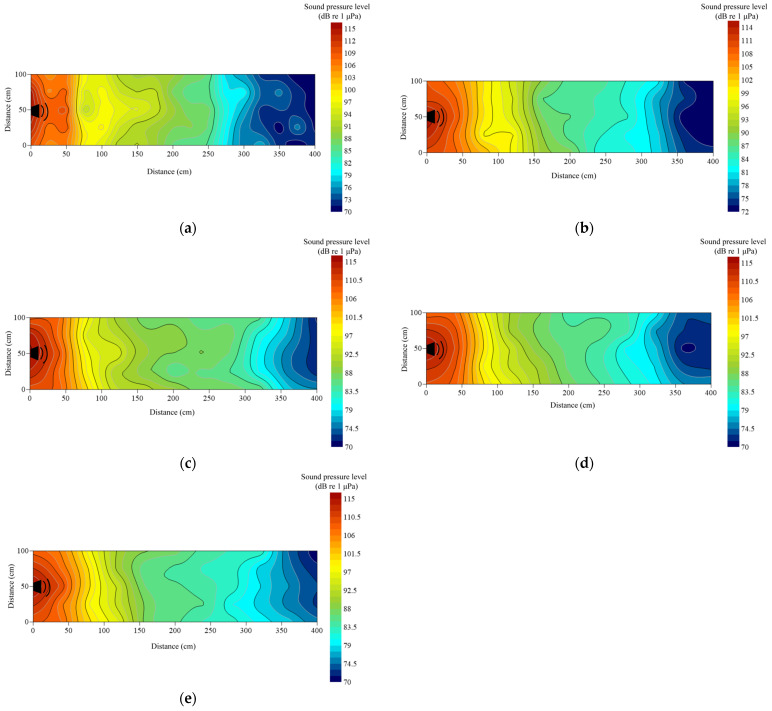
Contour maps of sound pressure level (dB re 1 μPa) of outboard motor noise with different PRIs. The measurement of sound intensity was made at a depth of 0.2 m at 400 positions in the cage during the playback of the sound stimulus. The intensity level was represented by different colors, marked on the scale on the right: (**a**) AS; (**b**) BS; (**c**) CS; (**d**) DS; (**e**) ES.

Outboard motor noise with different PRIs did not exhibit significant differences in sound pressure level attenuation. During playback of AS, attenuation occurred relatively rapidly within 1.2 m of the loudspeaker, with an overall reduction of approximately 38.48 dB re 1 μPa, indicating that the outboard motor decreased markedly over a short distance (Figure 7a). During playback of BS–ES, attenuation patterns similar to those of AS were observed, with SPL at the end of the net cage remaining around 72 dB re 1 μPa (Figure 7b–e). Overall, outboard motor noise with different PRIs showed relatively gradual longitudinal attenuation, whereas lateral attenuation was more rapid.

**Figure 8:**
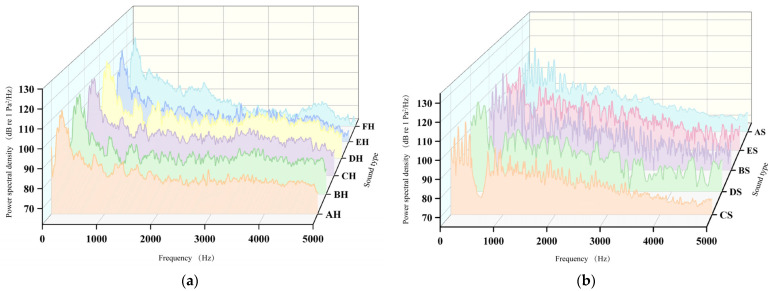
The power spectrum: (**a**) *Alligator sinensis hissing* with different PRIs and (**b**) outboard motor noise with different PRIs 3.3. Formatting of Mathematical Components.

Continuous *Alligator sinensis* hissing (AH) and *Alligator sinensis* hissing with different PRIs (BH–FH) showed high acoustic energy within 0–1000 Hz, with Power spectral density (PSD) decreasing progressively at higher frequencies, consistent with broadband characteristics. All sounds exhibited a pronounced peak around 100 Hz, indicating dominance of low-frequency components. In the 1000–3000 Hz range, CH, DH, and EH displayed similar fluctuation patterns, whereas AH was smoother and FH showed overall lower PSD. Across 3000–5000 Hz, SPLs declined further; however, BH and CH maintained noticeable fluctuations, suggesting more pronounced high-frequency components in intermittent vocalizations, while FH exhibited the lowest PSD in this band (Figure 8a).

Continuous outboard motor noise (AS) and outboard motor noise with different PRIs (BS–ES) also showed elevated PSD within 0–1000 Hz, peaking at 100–200 Hz. DS and CS exhibited reduced low-frequency energy, indicating that shorter pulse repetition frequencies weakened low-frequency components. In the 1000–3000 Hz range, DS and CS showed lower and more stable PSD distributions. Across 3000–5000 Hz, AS and ES maintained higher PSD than the other sounds. Overall, AS exhibited strong low- and mid-frequency SPL, whereas ES maintained relatively high PSD across all bands. In contrast, PSD of DS, CS, and BS progressively decreased within 0–3000 Hz (Figure 8b).

### 3.2. Response Frequency

**Figure 9:**
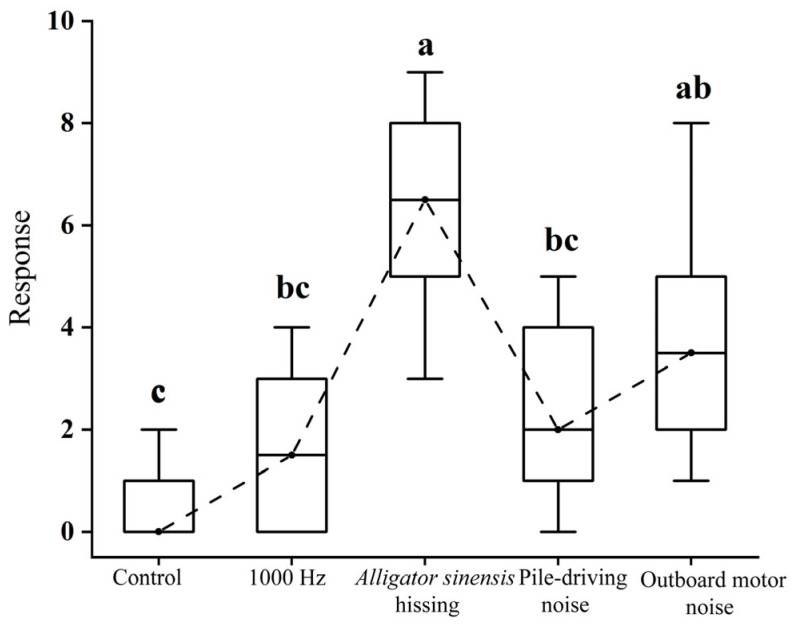
*Mean ± SD* response frequency of juvenile grass carp under different sound treatments. Different lowercase letters indicate significant differences (*p* < 0.05) among treatments following *Kruskal–Wallis* test.

Significant differences in the number of behavioral responses of juvenile grass carp were detected among sound treatments (*H* = 30.218, *p* < 0.05). Under control and during exposure to the 1000 Hz pure tone, mean response frequencies were 0.5 ± 0.71 and 1.6 ± 1.43, respectively. Although responses increased under pure tone exposure, the difference was not statistically significant compared with the control (*p* > 0.05). Playback of *Alligator sinensis* hissing and outboard motor noise elicited substantially higher response frequencies (6.30 ± 1.83 and 3.90 ± 2.23, respectively), both significantly exceeding control levels (*p* < 0.05). No significant difference was observed between the 1000 Hz pure tone and pile-driving noise treatments (*p* > 0.05) (Figure 9).

### 3.3. Total Midline Number of Crossings

**Figure 10:**
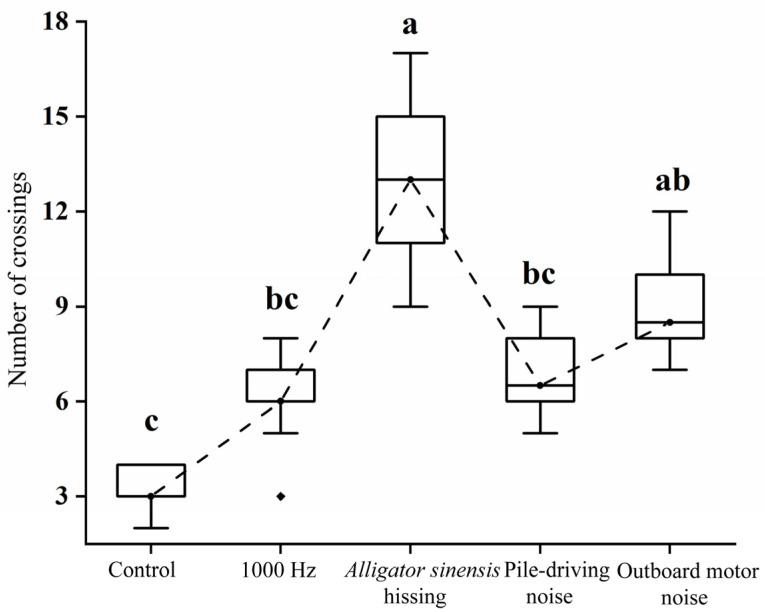
*Mean ± SD* total midline number of crossings of juvenile grass carp under different sound treatments. Different lowercase letters indicate significant differences (*p* < 0.05) among treatments following *Kruskal–Wallis* test.

Significant differences in the total number of midline crossings by juvenile grass carp were detected among treatments (*H* = 30.218, *p* < 0.05). Exposure to *Alligator sinensis* hissing elicited the highest crossing frequency, with a maximum of 15 events recorded. Under outboard motor noise, the mean number of crossings was 9.10 ± 1.60, with a maximum of 11 events. No significant difference was observed between these two broadband treatments (*p* > 0.05). Playback of 1000 Hz pure tone and pile-driving noise resulted in 6.10 ± 1.37 and 6.9 ± 1.37 crossings, respectively, both significantly higher than the control (*p* < 0.05), but not significantly different from each other (*p* > 0.05) (Figure 10). Overall, broadband sounds induced higher crossing frequencies than 1000 Hz pure tone stimulation. During 1000 Hz pure tone exposure, most individuals exhibited minimal behavioral change; some remained stationary, whereas others swam on the sound-source side of the cage.

### 3.4. First Response Time

**Figure 11:**
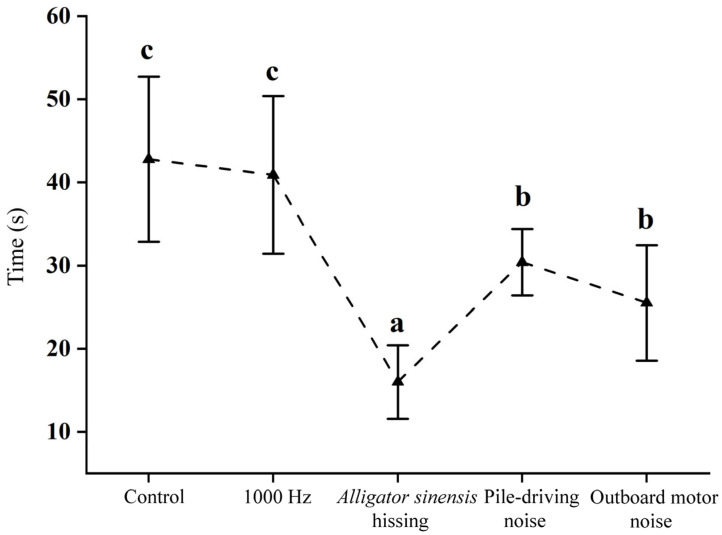
*Mean ± SD* first response time of juvenile grass carp under different sound treatments. Different lowercase letters indicate significant differences (*p* < 0.05) among treatments following one-way ANOVA test.

Significant differences in the first response time of juvenile grass carp were detected between the control and experimental groups under different sound stimuli (*F* = 23.307, *p* < 0.05). In the control, the mean first response time was 42.80 ± 9.92 s. Under 1000 Hz pure tone exposure, the time was 40.90 ± 9.48 s, showing no significant difference from the control (*p* > 0.05). Exposure to *Alligator sinensis* hissing elicited the shortest time, with a mean of 16.00 ± 4.42 s. Following playback of pile-driving noise and outboard motor noise, first response times were 30.40 ± 4.01 s and 25.50 ± 6.96 s, respectively. No significant difference was detected between these two treatments, but both were significantly shorter than those in the control and 1000 Hz pure tone groups (*p* < 0.05) (Figure 11).

### 3.5. Maximum Swimming Speed

**Figure 12:**
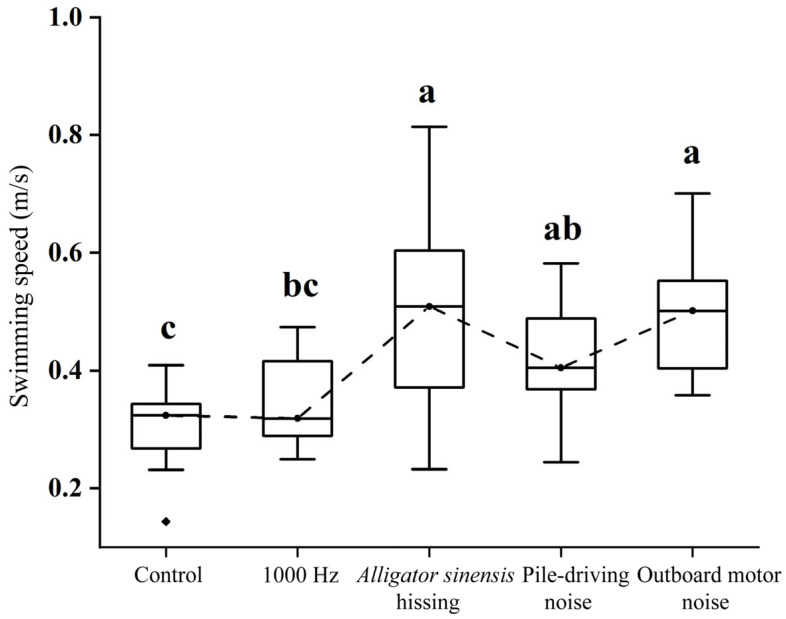
*Mean ± SD* maximum swimming speed of juvenile grass carp under different sound treatments. Different lowercase letters indicate significant differences (*p* < 0.05) among treatments following one-way ANOVA test.

Significant differences in maximum swimming speed were detected among treatments (*F* = 5.723, *p* < 0.05). The control group exhibited a maximum swimming speed of 0.30 ± 0.07 m/s, which did not differ significantly from that observed under 1000 Hz pure tone exposure (0.35 ± 0.07 m/s; *p* > 0.05). Exposure to *Alligator sinensis* hissing and outboard motor noise resulted in higher maximum swimming speeds (0.50 ± 0.19 m/s and 0.50 ± 0.11 m/s, respectively), both significantly greater than those recorded in the control and 1000 Hz treatments (*p* < 0.05). However, neither differed significantly from the pile-driving noise treatment (0.42 ± 0.11 m/s; *p* > 0.05) (Figure 12).

### 3.6. Average Response Speed

**Figure 13:**
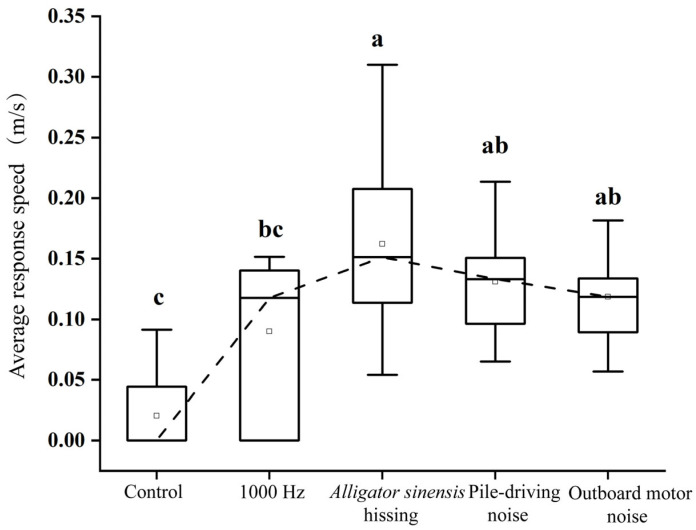
*Mean ± SD* average response speed of juvenile grass carp under different sound treatments. Different lowercase letters indicate significant differences (*p* < 0.05) among treatments following *Kruskal–Wallis* test.

Average response speed differed significantly among treatments (*H* = 22.16, *p* < 0.05). In the control group, juvenile grass carp remained largely stationary, exhibiting a low average response speed of 0.02 ± 0.03 m/s. Pile-driving noise and outboard motor noise were associated with increased response speeds (0.13 ± 0.04 m/s and 0.12 ± 0.04 m/s, respectively), both significantly higher than the control (*p* < 0.05), but not significantly different from the 1000 Hz pure tone treatment (*p* > 0.05). Exposure to *Alligator sinensis* hissing resulted in the highest mean response speed (0.16 ± 0.07 m/s), which did not differ significantly from the pile-driving or engine noise treatments (*p* > 0.05), but remained significantly higher than the control (*p* < 0.05) (Figure 13).

### 3.7. Swimming Behavior and Responses

**Figure 14:**
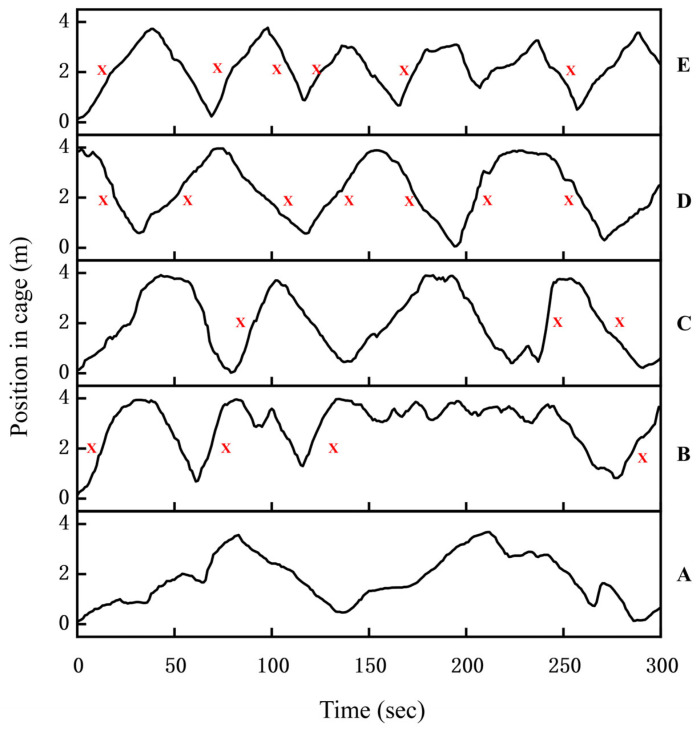
Motion process curve of grass carp to continuous and broadband sounds (N = 5). A Control, B 1000 Hz, C Pile-driving noise, D outboard motor noise, E *Alligator sinensis* hissing, 2. Red “X” represents the negative phonotactic response of the fish.

Representative curves illustrating the behavioral responses of juvenile grass carp to one pure tone, three broadband sounds, and the control treatment are presented (Figure 14). This figure depicts the position of juvenile grass carp along the lateral axis of the cage during the 5 min experimental period, with 0 on the *y*-axis corresponding to the end nearest the underwater speaker. In the control trials, juvenile grass carp exhibited free-swimming behavior in the absence of acoustic stimulation. In the control group without acoustic stimulation, juvenile grass carp swam slowly back and forth, with approximately 50% of individuals remaining stationary at times. Under 1000 Hz pure tone exposure, 20% of fish exhibited negative phonotaxis. During pile-driving noise playback, alternating sound sources induced back-and-forth swimming, but swimming speed remained low and the number of responses ranged from 2 to 3. In contrast, exposure to outboard motor noise and *Alligator sinensis* hissing elicited rapid movement in 95% of the fish during the first playback, with increased swimming speed and 6–8 responses, suggesting avoidance behavior.

### 3.8. Distribution Rate

**Figure 15:**
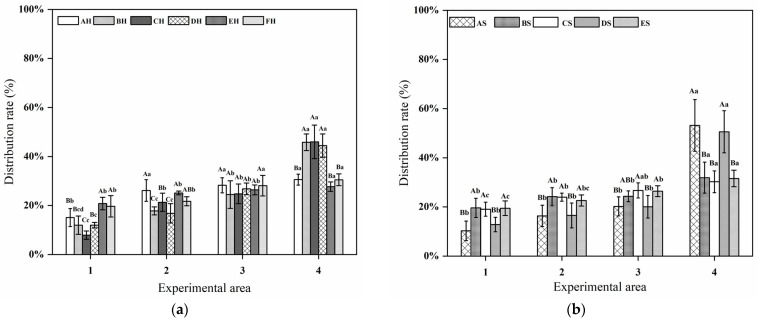
Distribution rate of juvenile grass carp: (**a**) under *Alligator sinensis* hissing with different PRIs and (**b**) under outboard motor noise with different PRIs. Different uppercase letters indicate significant differences among treatments, while different lowercase letters indicate significant differences (*p* < 0.05) among groups within the same treatment following one-way ANOVA test.

When juvenile grass carp were exposed to AH, the lowest distribution rate was observed in Area 1, while no significant differences were detected among the other areas (*p* > 0.05). Under BH exposure, the distribution rate in Area 4 was significantly higher than in the other three areas (*p* < 0.05). Similarly, during CH and DH treatments, fish showed significantly higher distribution rates in Area 4 compared with Areas 1–3 (*p* < 0.05), with the lowest proportion consistently recorded in Area 1. Under FH exposure, the highest distribution rate was also observed in Area 4 (30.50% ± 2.41%). Comparisons among different sound treatments within the same area revealed that, in Area 1, fish exposed to CH exhibited the lowest distribution rate. In Area 2, no significant differences were detected between AH and EH treatments (*p* > 0.05). In Area 3, distribution rates did not differ significantly among sound treatments (*p* > 0.05). In Area 4, no significant differences were found among BH, CH, and DH exposures (*p* > 0.05). Overall, exposure to these sound treatments resulted in a spatial shift away from the sound source. The highest distribution rate in Area 4 occurred under CH exposure, indicating the strongest negative phonotactic response among the tested sounds (Figure 15a).

When juvenile grass carp were exposed to AS, the distribution rate in Area 4 was significantly higher than in the other areas (*p* < 0.05). Under BS exposure, the proportion of fish in Area 4 was also markedly higher than in the remaining areas. During CS treatment, no significant difference was detected between Areas 4 and 3 (*p* > 0.05), whereas the distribution rate in Area 2 was significantly higher than in Area 1 (*p* < 0.05). Under DS and ES exposures, the distribution rates in Area 4 were 50.57% ± 8.56% and 31.56% ± 3.37%, respectively, both significantly higher than those in the other areas (*p* < 0.05). Comparisons among sound treatments within the same area showed that, in Area 1, distribution rates under BS and CS were significantly higher than under the other treatments (*p* < 0.05), whereas AS resulted in the lowest proportion. In Area 2, fish exposed to DS exhibited a significantly lower distribution rate than those under BS and CS (*p* < 0.05). In Area 4, no significant difference was detected between AS and DS (*p* > 0.05), and both treatments yielded higher distribution rates than the other conditions. Overall, exposure to AS and DS was associated with reduced occupancy in Area 1 and increased distribution in Area 4, indicating a pronounced negative phonotactic response (Figure 15b).

### 3.9. The Selection Coefficient

**Figure 16:**
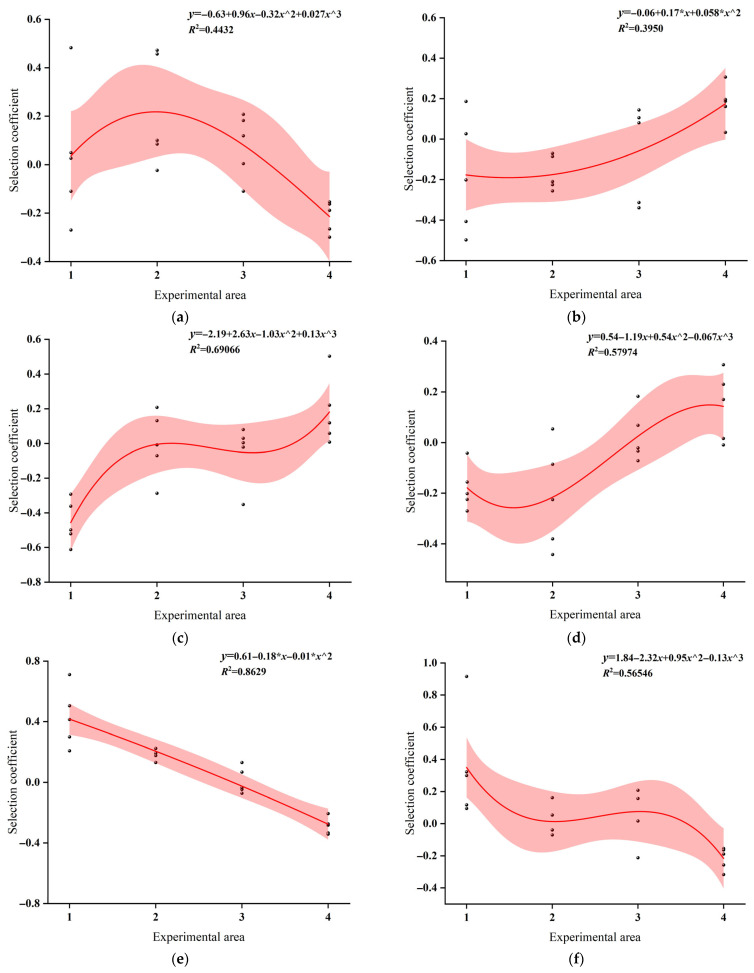
The selection coefficient of Alligator sinensis hissing with different PRIs: (**a**) AH; (**b**) BH; (**c**) CH; (**d**) DH; (**e**) EH; (**f**) FH.

Regression analysis was performed using the experimental area as the independent variable and the selection index of juvenile grass carp as the dependent variable. All treatments were conducted at the same SPL. Under AH exposure, the selection index increased from Area 1 to Area 2, indicating a relative preference for Area 2 (Figure 16a). However, the overall regression trend showed a gradual decline, suggesting a greater preference in areas closer to the sound source. During EH exposure, the regression line exhibited a decreasing trend. Negative selection index values indicated avoidance of the corresponding areas (Figure 16e). Under FH treatment, the selection index increased slightly from Area 2 to Area 3, followed by a marked decline toward Area 4, indicating a relative preference for Area 3 and avoidance of Area 4 (Figure 16f). When exposed to BH, the regression trend increased progressively, indicating stronger avoidance in areas closer to the speaker, with avoidance intensity increasing as distance to the speaker decreased, while Areas 3 and 4 were relatively preferred (Figure 16b). In contrast, under CH and DH exposures, the selection index first decreased and then increased along the spatial gradient (Figure 16c,d). Preference increased with distance from the speaker, whereas Areas 1 and 2 were avoided.

**Figure 17:**
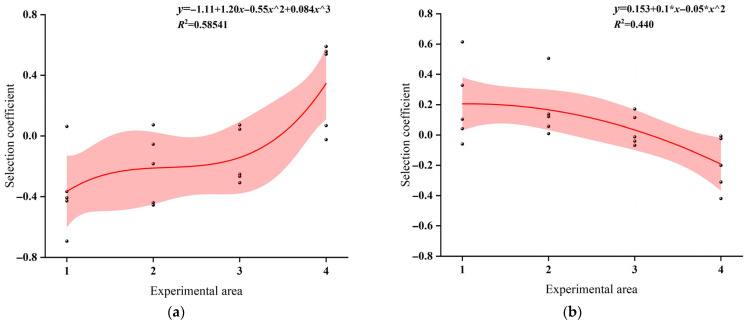

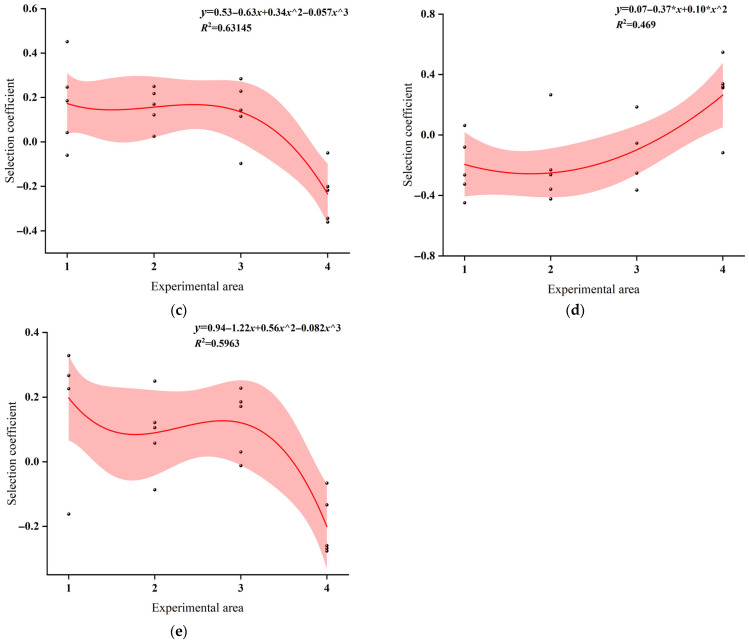
The selection coefficient of outboard motor noise with different PRIs: (**a**) AS; (**b**) BS; (**c**) CS; (**d**) DS; (**e**) ES.

Under AS and DS exposures, the regression lines showed a gradual increasing trend, with stronger avoidance observed near the sound source and greater preference for Area 4 as sound intensity decreased (Figure 17a,d). During BS and CS treatments, the regression lines showed decreasing trends, with the highest selection indices in Area 1, indicating a relative preference for this area (Figure 17b,c). Under ES exposure, the selection index increased from Area 2 to Area 3 and then declined toward Area 4 (Figure 17e). Negative selection index values indicated avoidance of the corresponding areas. For DS exposure, negative selection indices were observed in Areas 1 and 2, reflecting pronounced negative phonotactic behavior and reduced swimming activity in these areas (Figure 17d). In contrast, under BS exposure, juvenile grass carp mostly remained stationary or swam slowly along the cage walls.

## 4. Discussion

### 4.1. Differences Between Semi-Natural Conditions and Indoor Enclosed Tanks

Acoustic deterrence has been widely reported to influence fish migration behavior in controlled laboratory environments [16]. *H. molitrix* and *A. nobilis* exhibited significant negative phonotaxis in response to outboard motor noise (0.06–10 kHz) in an outdoor concrete tank [12,16]. Similarly, *Ptychobarbus kaznakovishowed* showed clear avoidance behavior in response to *Alligator sinensis* hissing sounds (0.05–5000 Hz) in an outdoor fiberglass tank [13]. However, most previous studies have been conducted in enclosed indoor tanks, where acoustic conditions are strongly affected by boundary reflections and standing waves. These conditions can produce localized resonance and highly heterogeneous sound pressure distributions, potentially limiting the ecological relevance of the observed behavioral responses [14]. In contrast, our findings from semi-natural net cage experiments in a reservoir-like open-water environment suggest that fish responses to acoustic stimuli may differ substantially under more realistic propagation conditions [33]. By reducing boundary reflections, the open-water setup allows sound to propagate more uniformly across the experimental area, thereby providing a more ecologically relevant assessment of acoustic deterrent effectiveness [33]. This difference helps to bridge the gap between laboratory-based conclusions and field applications, and improves our understanding of how acoustic barriers may function under real-world hydrodynamic and acoustic conditions. In addition to these environmental differences, it is important to recognize that acoustic propagation in shallow water is subject to physical constraints such as cutoff frequency [33]. In the present study, although the nominal cutoff frequency based on the cage depth (the water depth inside the cage was approximately 0.4 m, the distance from the bottom of the cage to the bottom of the underlying natural water body was approximately 0.8 m, and the total water depth was approximately 1.2 m) was estimated to be approximately 312.5 Hz, the additional water depth beneath the cage likely facilitated partial transmission of lower-frequency components [34]. Moreover, reflections, scattering, and interference may have created a complex sound field in which even frequencies near or below the theoretical cutoff could still be detected locally by the fish [35].

In the present study, pile-driving noise and outboard motor noise exhibited similar magnitudes of lateral and longitudinal attenuation, with rapid decay occurring at approximately 1 m from the loudspeaker (Figure 4c,d). In contrast, *Alligator sinensis* hissing showed the weakest attenuation in water; as distance increased, the rate of lateral attenuation gradually decreased, whereas longitudinal attenuation remained relatively rapid (Figure 4). Under natural aquatic conditions, sound propagation and attenuation are governed by a combination of environmental and geoacoustic properties. Increased turbidity can enhance acoustic scattering due to suspended particulate matter, thereby increasing transmission loss, particularly at higher frequencies where scattering effects are more pronounced [36]. These processes can result in highly non-uniform sound fields and spatially variable attenuation patterns. Furthermore, sediment type plays a critical role in bottom-related acoustic losses. Muddy sediments typically exhibit higher acoustic absorption and lower impedance contrast with the overlying water, resulting in stronger energy dissipation. In contrast, sandy substrates tend to enhance bottom reflection and support multi-path propagation, which may increase spatial variability in received sound levels [37]. Collectively, these environmental factors contribute to substantial variability in underwater sound fields, which should be considered when interpreting fish behavioral responses under natural conditions.

Compared with the sound field distributions reported in previous studies [17,38], sound attenuation in the present study occurred more rapidly. This difference due to the open-water experimental conditions used in this study. In indoor tank experiments, sound waves are repeatedly reflected by tank boundaries, which can lead to an accumulation of reflected energy and a slower apparent attenuation rate [13]. This difference also due to both the open-water experimental conditions and the substrate characteristics of the study environment. In the present study, the bottom consisted primarily of soft sediment (e.g., mud), which exhibits higher acoustic absorption and lower acoustic impedance contrast with the overlying water column [37]. As a result, a greater proportion of acoustic energy is dissipated into the sediment, leading to enhanced transmission loss. In contrast, previous studies conducted in controlled tank environments or hard-bottom conditions (e.g., concrete or glass tanks) typically involve stronger boundary reflections. In indoor tank experiments, sound waves are repeatedly reflected by rigid boundaries, leading to an accumulation of reflected energy and the formation of standing wave fields, which can sustain acoustic energy within the water column and result in a slower apparent attenuation rate [35]. The observed spatial heterogeneity of the sound field may appear somewhat unexpected, given that loudspeakers are generally assumed to exhibit relatively stable directional radiation patterns. However, in practical aquatic environments, the effective sound field is not solely determined by source directivity but is strongly influenced by propagation conditions [38]. In open-water settings, interactions between emitted sound waves and environmental boundaries, including the water surface and bottom substrate, can produce partial reflections and interference patterns [38]. In addition, the presence of cage structures may introduce scattering and diffraction effects, further distorting the local sound field [39]. Spatial variability in water depth and environmental heterogeneity can also alter propagation pathways and redistribute acoustic energy [40]. As a result, multi-path propagation and boundary interactions may generate localized fluctuations in sound pressure levels and non-uniform attenuation patterns [41]. Therefore, the spatial heterogeneity observed in this study should be interpreted as an emergent property of a complex shallow-water acoustic environment, rather than as an inconsistency in the performance of the sound source.

By contrast, the experimental net cages in this study were deployed in a natural water body, allowing sound to propagate beyond the cage over a wider area. As a result, although the propagation range was broader, sound attenuated more rapidly, while boundary effects on the experimental results were effectively reduced. Previous indoor studies have demonstrated that fish exhibit strong avoidance responses to acoustic stimuli, often characterized by high response frequencies, short first response times, and maximum swimming speed [12,13,17]. In the present study, juvenile *C. idella* exposed to three different types of broadband sounds also exhibited clear negative phonotaxis. However, their response frequency, average swimming speed, maximum swimming speed, total midline number of crossings, and first-response time differed in certain aspects from those reported in indoor experiments [42,43,44]. These differences likely reflect the more complex environmental conditions and more realistic sound propagation patterns in natural waters [44]. In addition to environmental complexity, differences in acoustic stimulus characteristics may also contribute to the observed behavioral discrepancies [44]. The outboard motor noise, *Alligator sinensis* hissing, and pile-driving sounds used in the present study are all broadband signals with pronounced temporal variability. Nevertheless, spectrographic analysis (Figure 5) indicates that these signals share a common feature, with relatively high energy concentrated within the 0.05–800 Hz frequency range. This frequency band overlaps with the known auditory sensitivity range of cyprinid fishes equipped with a Weberian apparatus, which is particularly sensitive to low- and mid-frequency acoustic cues [23]. Therefore, the observed behavioral responses are likely influenced not only by environmental factors but also by the spectral–temporal characteristics of the acoustic stimuli themselves, which determine their perceptual salience to the test species. Notably, despite slightly lower response magnitudes compared with indoor studies, the observed behaviors are likely to more accurately represent the natural sensitivity of *C. idella* to acoustic stimuli, thereby enhancing the ecological relevance of the findings for the application of acoustic deterrent strategies.

Nevertheless, the present study has some limitations. The semi-enclosed experimental setup could not completely eliminate boundary effects, and the decision not to use fully open cages was made to allow clearer observation of behavioral indicators. Therefore, while cutoff frequency provides a useful theoretical reference for interpreting acoustic constraints, it should be considered alongside the specific physical characteristics of the experimental system. Future studies should aim to directly measure the acoustic field within such systems, including frequency-dependent attenuation and spatial heterogeneity, and further examine fish behavioral responses under fully open-water conditions to improve the robustness and applicability of acoustic deterrent strategies.

### 4.2. Impact of Acoustic Stimuli on Grass Carp Negative Phonotaxis

Sound type is a key factor influencing fish avoidance behavior in response to acoustic stimuli [32]. For example, Yang et al. (2024) [43] reported that juvenile *C. idella* exhibited the highest mean response frequency when exposed to *Alligator sinensis* roar. Similarly, Vetter et al. (2017) [16] found that *H. molitrix* showed the highest response frequency under outboard motor noise. He et al. (2024) [44] further reported that juvenile *C. idella* displayed the highest response frequency and phonotactic speed, as well as the shortest first-response time, when exposed to yacht noise. In the present study, juvenile *C. idella* exposed to *Alligator sinensis* hissing and outboard motor noise exhibited response frequencies of 6.30 ± 1.83 and 3.90 ± 2.23, respectively, both significantly higher than those of the control group (Figure 9). Among the tested sound stimuli, the shortest first-response time was observed under *Alligator sinensis* hissing (16.00 ± 4.42 s). In contrast, exposure to pile-driving noise and outboard motor noise resulted in first response times of 30.40 ± 4.01 s and 25.50 ± 6.96 s, respectively, both significantly shorter than those observed in the control group and under the 1000 Hz pure tone treatment (Figure 11). These findings are consistent with previous studies by Yang et al. (2024) [43] and He et al. (2024) [44], indicating that juvenile grass carp may respond to *Alligator sinensis* hissing and outboard motor noise with negative phonotaxis across both indoor and semi-natural settings. However, such responses should be interpreted with caution, as they are likely influenced by environmental context and may vary under different acoustic and habitat conditions. Further validation under a wider range of environmental scenarios is therefore required.

In the present study, when juvenile *C. idella* were exposed to *Alligator sinensis* hissing and outboard motor noise, they exhibited strong negative phonotaxis (Figure 14). This negative phonotaxis behaviour was influenced not only by the intensity and frequency of the test sounds but also by the biological significance conveyed by the acoustic signals. Fish often display avoidance behavior in response to sounds produced by predators [13], outboard motor noise [12], and underwater blasting [32]. Our results showed that juvenile *C. idella* were more sensitive to low-frequency sound waves. This enhanced response is consistent with the known auditory characteristics of fish, particularly their sensitivity to particle motion [35]. In shallow-water and near-field environments, particle motion often serves as a dominant cue for sound detection, especially at low frequencies where auditory thresholds are generally lower [45]. As a cyprinid fish, *C. Idella* possesses a Weberian apparatus, which is associated with a relatively broad hearing frequency range and lower auditory thresholds, thereby enhancing auditory sensitivity to acoustic stimuli [23]. Some cyprinid fishes are capable of detecting sounds above 3000 Hz, and *H. molitrix* and *Aristichthys nobilis* can perceive a broad frequency range of approximately 0.1–5.0 kHz [12,16]. Moreover, the strongest negative phonotactic response was observed when juvenile *C. idella* were exposed to *Alligator sinensis* hissing. This may be because *Alligator sinensis* hissing contains acoustic characteristics similar to those produced by aquatic predator activity, which may be perceived by juvenile *C. idella* as potential danger signals, triggering rapid avoidance behavior [46,47]. In addition, outboard motor noise is characterized by continuous low-frequency sound waves, which may interfere with the vestibular system of *C. idella*, causing discomfort or a sense of imbalance and thereby driving them to move away from the sound source [48]. To enhance the long-term effectiveness of acoustic deterrence and reduce potential habituation, future applications could incorporate adaptive playback strategies, such as variable-frequency signals or randomized temporal intervals, which may help maintain stimulus novelty and sustain avoidance responses over time.

### 4.3. Effects of Temporal Structure on Sound Field Distribution and Frequency Characteristics

The cumulative sound exposure level (SEL*_cum_*) of short pulses results in a more concentrated energy distribution within the sound field, leading to higher sound pressure levels; in contrast, sound fields generated by long pulses are more dispersed, primarily due to longer intervals between pulses and potentially lower sound pressure levels of individual pulses [49]. Differences among CH, DH, and EH were mainly reflected in subtle fluctuations within the mid- and high-frequency bands. As the pulse repetition interval increased, sound pressure levels gradually decreased, with part of the acoustic energy redistributed to other frequency bands. FH exhibited the lowest sound pressure level within the 3000–5000 Hz range, suggesting that randomly impulsive sounds have a stronger influence on high-frequency components and reduce acoustic energy in this band (Figure 8a). In contrast, AS exhibited relatively strong sound pressure levels in both the low and mid frequency bands, while ES maintained comparatively high sound pressure levels across all frequency ranges. Continuous or randomly impulsive signals retained more acoustic energy at higher frequencies. By comparison, sound pressure levels of BS, CS, and DS decreased sequentially within the 0–3000 Hz band, indicating that longer pulse intervals result in greater energy loss (Figure 8b). Notably, signals with irregular pulse intervals may enhance stimulus salience compared to temporally regular signals, as temporal structure has been shown to influence fish behavioral responses to acoustic stimuli [50,51]. Such unpredictability may increase perceived risk and maintain heightened vigilance in fish, thereby reducing the likelihood of rapid habituation [52]. In contrast, regularly repeated signals may become predictable over time, allowing fish to gradually attenuate their behavioral responses through habituation [52].

### 4.4. Impact of Temporal Patterns of Acoustic Signals on Fish Behavior

The temporal structure of acoustic signals plays a critical role in shaping fish behavioral responses [31,53]. Our results indicate that impulsive sounds with different pulse repetition intervals (PRIs) can alter swimming patterns and potentially modulate vigilance during acoustic exposure. When juvenile grass carp were exposed to AH, EH, FH, BS, CS, and ES treatments, the selection coefficient generally decreased, with higher values observed in areas closer to the loudspeaker. In contrast, BH, CH, DH, AS, and DS treatments produced an overall increasing trend in the selection coefficient, although this response may vary under different environmental or acoustic contexts. The observed spatial redistribution indicates a consistent movement away from the sound source, with fish progressively aggregating in areas farther from the acoustic origin. This pattern is consistent with negative phonotaxis, a well-documented response in fish exposed to anthropogenic noise [51,52]. However, the magnitude of this response varied markedly among treatments, indicating that spatial behavior was not solely determined by distance from the sound source but was strongly influenced by acoustic signal structure. In particular, CH and DS treatments elicited stronger avoidance responses (Figure 15), characterized by reduced occupancy near the sound source and increased aggregation in distal areas. These differences are likely associated with temporal acoustic features, including pulse repetition interval and signal predictability, which can influence stimulus salience, vigilance, and perceived environmental risk [31,52]. Accordingly, reduced occupancy near the sound source likely reflects enhanced avoidance behavior rather than simple spatial preference. From an ecological perspective, this spatial redistribution reflects a trade-off between habitat use and perceived risk under acoustic disturbance. Areas close to the sound source may represent higher-risk zones, whereas more distant areas may function as behavioral refuges. Similar refuge-seeking strategies have been widely documented in fish exposed to anthropogenic noise, where individuals adjust spatial positioning to reduce exposure while maintaining access to essential resources [26].

Importantly, the underlying mechanism of this avoidance behavior may not be unitary. On one hand, the observed avoidance of Areas 1 and 2 may reflect a non-specific stress response to elevated broadband anthropogenic noise. Anthropogenic acoustic disturbance has been shown to induce generalized arousal, increase vigilance costs, disrupt spatial behavior, and reduce habitat attractiveness, ultimately leading to broad-scale displacement from high-exposure zones [51,54]. From this perspective, the spatial avoidance likely represents a generalized disturbance response driven by physiological and behavioral stress rather than stimulus-specific processing. On the other hand, the spatial pattern may also involve a more specific risk-related interpretation of acoustic cues. Certain impulsive acoustic features may resemble naturally occurring environmental signals associated with disturbance or predation risk. Although grass carp are not expected to possess evolutionary memory of anthropogenic noise, fish auditory systems are capable of generalizing across acoustic cues sharing abrupt onset, temporal irregularity, and high unpredictability—features commonly associated with biologically relevant threat signals in natural environments [55,56]. This may lead to innate or experience-dependent risk-assessment processes that functionally resemble predator recognition in behavioral output. Although the present study cannot definitively separate these two mechanisms, the stronger avoidance observed in Areas 1 and 2 suggests that fish responses are not solely driven by spatial proximity or broadband masking effects. Instead, they likely involve higher-order processing of acoustic salience and risk relevance. Similar dual-pathway interpretations, integrating generalized stress responses and stimulus-specific risk perception, have been proposed in studies of fish responses to anthropogenic noise and environmental stressors [52]. Finally, the stronger responses observed under CH and DS further indicate that not all acoustic signals are behaviorally equivalent. Certain temporal structures may enhance detectability and reinforce avoidance, whereas others may allow partial acclimation through habituation processes. This highlights temporal predictability as a key factor shaping behavioral sensitivity to repeated acoustic exposure.

Future work combining controlled acoustic playback experiments with physiological stress indicators (e.g., cortisol measurements) and habituation paradigms will be necessary to disentangle generalized stress responses from stimulus-specific risk interpretation mechanisms, and to better resolve the cognitive basis of noise-induced spatial avoidance in fish.

## 5. Conclusions

This study demonstrates that juvenile *C. idella* exhibited pronounced negative phonotaxis in response to *Alligator sinensis* hissing and outboard motor noise, suggesting that these sound types may function as potential acoustic deterrent cues. Variations in pulse characteristics appeared to influence fish phonotactic behavior, highlighting the potential importance of temporal structure in acoustic exposure. However, because the experiments were conducted under semi-natural conditions, the results should be interpreted with caution when extrapolating to natural environments. Several methodological limitations should be acknowledged.

First, although the experimental enclosure was located in a reservoir-like environment, acoustic reflections from tank boundaries and surrounding shorelines may have introduced spatial heterogeneity in the sound field, potentially affecting fish distribution patterns. Second, the influence of water depth on behavioral responses was not explicitly tested in this study. Third, and importantly, only sound pressure levels were measured, whereas particle motion—the primary acoustic component detected by many fish species—was not quantified. The absence of particle motion measurements represents a critical limitation, as it may restrict accurate interpretation of auditory perception and sensitivity in the tested species. In addition, the physiological consequences of the observed behavioral responses remain unclear. Therefore, the present findings should be considered as preliminary evidence indicating potential effects of different pulse repetition intervals (PRIs) on fish behavior, rather than definitive conclusions regarding deterrent efficacy.

Future research should further investigate additional temporal parameters, including pulse repetition regularity, pulse duration, and pulse shape, to improve the design and effectiveness of acoustic deterrent systems for invasive fish management. Moreover, future studies should incorporate measurements of particle motion alongside sound pressure levels to better characterize the acoustic field experienced by fish. It is also recommended to integrate physiological validation approaches, including assessments of key auditory and mechanosensory structures such as the swim bladder, lateral line system, and hair cells, as well as histological or tissue-level analyses before and after acoustic exposure, to more comprehensively evaluate the biological impacts of acoustic stimuli.

## Data Availability

The data analyzed in this study are available from the corresponding author upon reasonable request.
